# Psychological Impact of the COVID-19 Pandemic on Chinese Health Care Workers: Cross-Sectional Survey Study

**DOI:** 10.2196/23125

**Published:** 2021-01-20

**Authors:** Jie Ni, Fang Wang, Yihai Liu, Mingyue Wu, Yan Jiang, Yujie Zhou, Dujuan Sha

**Affiliations:** 1 General Medical Department Nanjing Drum Tower Hospital the Affiliated Hospital of Nanjing University Medical School Nanjing China; 2 Department of Emergency Nanjing Drum Tower Hospital the Affiliated Hospital of Nanjing University Medical School Nanjing China; 3 Department of Cardiology Nanjing Drum Tower Hospital Clinical College of Nanjing Medical University Nanjing China; 4 Department of Education Nanjing Drum Tower Hospital the Affiliated Hospital of Nanjing University Medical School Nanjing China

**Keywords:** 2019-nCoV, COVID-19, frontline clinician, medical students, psychology

## Abstract

**Background:**

The outbreak of COVID-19 has dominated headlines worldwide. The number of infections has continued to rise and had reached 30,000 worldwide at the time this paper was written. Because of the high risk of nosocomial transmission, medical health care workers may be experiencing substantial psychological stress. This descriptive study aimed to identify psychosocial effects on hospital staff associated with working in a hospital environment during the COVID-19 outbreak.

**Objective:**

Our survey participants included 57 frontline clinicians working at Wuhan First Hospital and 157 medical students working at Jiangsu Provincial People’s Hospital during the COVID-19 outbreak. The questionnaire we adopted included questions regarding the participants’ personal well-being, sociodemographic characteristics, and psychological status.

**Methods:**

57 frontline clinicians working in Wuhan First Hospital and 157 medical training students working in Jiangsu Provincial Peoples Hospital during this outbreak participated in our survey. The questionnaire we adopted included questions regarding the participants’ personal well-being, sociodemographic characteristics and the psychological status.

**Results:**

The COVID-19 outbreak had psychological impacts both on formal workers and medical students. The psychological effects included sleep disorders, anxiety, and depression. There was no significant difference between the group of formal workers and medical students (*P*=.85), and more than 50% (30/54, 56%, vs. 83/157, 52.9%) of the respondents reported pandemic-related mental disorders.

**Conclusions:**

Our study indicates that the high risk of SARS-CoV-2 exposure caused substantial psychological stress among health care workers. This finding emphasizes the need to promote psychological crisis intervention for medical personnel during this epidemic.

## Introduction

A novel pneumonia associated with a coronavirus broke out suddenly in Wuhan, China in December 2019 [1]. The Chinese government reported that approximately five million residents left Wuhan and traveled to other provinces within China, while thousands of people reached other countries before the lockdown. The number of infections continued to rise and had reached 30,000 at the time of the writing of this paper according to real-time data on Weibo.com [2]. Along with the rapid expansion of the number of patients, health care systems worldwide were faced with difficult predicaments, including severe shortages of health care workers and medical materials [3]. Furthermore, health care providers had a greater likelihood of being exposed to the virus [4]. Research on the impact of previous epidemic outbreaks on the psychological well-being of health care workers showed that many health care workers presented high levels of psychological distress [5]. Although the Chinese government endeavored to guarantee the security of frontline clinicians, including organizing strict training, providing adequate medical facilities, and discouraging off-work contact, little is known about the psychological effects of the COVID-19 outbreak on hospital workers. The main objective of our study was to investigate whether medical personnel were experiencing significant psychological conflict between their duties and their concern for their own safety [6] and to evaluate whether psychological intervention was necessary.

## Methods

### Sample

This was a cross-sectional study including 57 frontline clinicians working at Wuhan First Hospital and 157 medical students working at Jiangsu Provincial People’s Hospital in Nanjing during the COVID-19 outbreak. First, we distributed the web-based questionnaire among the Wuhan First Hospital frontline staff, and we collected 57 valid questionnaires on March 4, 2020. Later, we conducted a survey among young medical students to investigate their psychological changes associated with the COVID-19 pandemic, and we collected 157 valid surveys. The questionnaire administered to the frontline clinicians consisted of 4 main sections: basic demographic data, the Athens Insomnia Scale, the Self-Rating Anxiety Scale (SAS), and the Self-Rating Depression Scale (SDS). The questionnaire administered to the medical students did not contain the Athens Insomnia Scale. Owing to resource constraints, the distribution of the questionnaire was limited to only those who received the questionnaire on day 2 of data collection. We coded the response categories using the scoring method recommended by Goldberg and Williams [7] and calculated a total score. We used a threshold score of greater than 50 to identify the presence of emotional anxiety and a threshold score of greater than 53 to identify the presence of emotional depression.

### Procedures

The questionnaires were completed on a voluntary basis by all willing respondents (physicians, nurses, and medical students working in hospitals during the crisis). We retrieved the completed questionnaires at a computer terminal. Reminders to complete the survey were sent to the volunteers by email.

### Ethics Approval and Consent

The studies involving human participants were reviewed and approved by the Ethics Committee of Nanjing Drum Tower Hospital. Written informed consent to participate in this study was provided by the participants.

### Statistical Analysis

We analyzed the data using SPSS 25.0 (IBM Corporation). Descriptive statistics were employed to organize the data collected from the survey. Chi-square tests were performed to compare the differences between groups. *P* values <.05 were considered statistically significant.

## Results

### Demographics

#### Wuhan Frontline Clinicians

A total of 54 physicians and nurses completed our survey voluntarily. The 54 respondents comprised 45 women (83%) and 9 men (17%) (Table 1). Among these medical staff, 26/54 (48%) were single, 26/54 (48%) were married, and 2/54 (4%) were divorced. Most of the 54 respondents were nurses (n=45, 83%), and physicians represented 17% of the sample. Among the physicians, 2 (22%) held senior professional titles; 6 (67%) held middle titles, and 1 (11%) held a junior title. Among the nurses, 2 (4%) had senior professional titles; 3 (7%) held middle titles, and 40 (89%) held junior titles. In this sample, 12/54 respondents (22%) had a master’s degree or above, and 42/54 (78%) had a bachelor’s or associate degree.

**Table 1:** Demographic characteristics of the frontline clinicians at Wuhan First Hospital (N=54).

Characteristic		Respondents with mental disorders (n=30), n (%)	Respondents without mental disorders (n=24), n (%)	*P* value
**Sex**				.07
	Male	2 (7)	7 (29)	
	Female	28 (93)	17 (71)	
**Age (years)**				.75
	20-30	14 (47)	11 (46)	
	30-40	14 (47)	10 (42)	
	≥40	2 (7)	3 (13)	
**Education**				.44
	College or bachelor’s degree	25 (83)	17 (71)	
	Master’s degree or above	5 (17)	7 (29)	
**Occupation**				.27
	Nurse	27 (90)	18 (75)	
	Physician	3 (10)	6 (25)	
**Tenure in current occupation (years)**				.76
	≤5	12 (40)	9 (38)	
	5-15	16 (53)	12 (50)	
	≥15	2 (7)	3 (13)	
**Employment title**				.44
	Junior	23 (77)	17 (71)	
	Middle	6 (20)	4 (17)	
	Senior	1 (3)	3 (13)	
**Marital status**				.95
	Single	14 (47)	12 (50)	
	Married	15 (50)	11 (46)	
	Divorced	1 (3)	1 (4)	

#### Nanjing Clinical Medicine Students

After excluding respondents who had a history of anxiety, depression, or sleep disorders, we included 157 clinical medicine students in our study. From the received data, we found that none of the participants in our study had any symptoms of COVID-19 or a history of direct contact with SARS-CoV-2.

### Sleep and Psychological Status

In contrast with the Nanjing clinical medicine students, Wuhan frontline clinicians were asked to complete an additional examination item: the Athens Insomnia Scale. In the group of frontline clinicians, the anxiety-related analysis showed that 9% (5/54) of participants had mild anxiety. Regarding the depression-related results, 35% (19/54) of the medical staff had mild depression, and 7% (4/54) had moderate depression. Synthesizing the results overall, we found that working experience, which was represented by work life, job title, and age, was highly associated with the survey results. People with more work experience had lower anxiety and depression rates. The results also showed that mental disorders were more common in nurses (Table 1).

When the risk of psychological symptoms was analyzed in a subsample of Nanjing clinical medicine students, we calculated that 47.1% (74/157) had mild depression and 1.2% (2/157) had moderate depression. Regarding the anxiety aspect, 4.0% (6/157) generally had mild depression. The distributions of the scores on the SAS and SDS for the medical students who participated in the study are shown in Table 2 and Table 3, respectively.

The high risk of psychological problems among health care providers observed in this study should evoke concern among psychologists.

**Table 2:** The distribution of the Self-Rating Anxiety Scale scores of the surveyed medical students (N=157).

Symptom	Score, n (%)			
	1	2	3	4
Anxiousness	100 (63.7)	50 (31.8)	3 (1.9)	4 (2.5)
Fear	128 (81.5)	24 (15.3)	2 (1.3)	3 (1.9)
Panic	125 (79.6)	27 (17.2)	3 (1.9)	2 (1.3)
Mental disintegration	142 (90.4)	14 (8.9)	1 (0.6)	0 (0)
Apprehension	68 (43.3)	15 (9.6)	23 (14.6)	51 (32.5)
Tremors	151 (96.2)	5 (3.2)	0 (0)	1 (0.6)
Body aches and pains	124 (79.0)	23 (14.6)	10 (6.4)	0 (0)
Easy fatiguability, weakness	108 (68.8)	42 (26.8)	6 (3.8)	1 (0.6)
Restlessness	50 (31.8)	11 (7.0)	34 (21.7)	62 (39.5)
Palpitation	120 (76.4)	30 (19.1)	5 (3.2)	2 (1.3)
Dizziness	141 (89.8)	15 (9.6)	1 (0.6)	0 (0)
Faintness	151 (96.2)	6 (3.8)	0 (0)	0 (0)
Dyspnea	100 (63.7)	8 (5.1)	7 (4.5)	42 (26.8)
Paresthesia	150 (95.5)	6 (3.8)	0 (0)	1 (0.6)
Nausea and vomiting	126 (80.3)	27 (17.2)	4 (2.5)	0 (0)
Urinary frequency	132 (84.1)	21 (13.4)	4 (2.5)	0 (0)
Sweating	83 (52.9)	19 (12.1)	18 (11.5)	37 (23.6)
Face flushing	136 (86.6)	18 (11.5)	2 (1.3)	1 (0.6)
Insomnia	38 (24.2)	14 (8.9)	38 (24.2)	67 (42.7)
Nightmares	111 (70.7)	44 (28.0)	2 (1.3)	0 (0)

**Table 3:** The distribution of the Self-Rating Depression Scale scores of the surveyed medical students (N=157).

Symptom	Score			
	1	2	3	4
Feeling downhearted	123 (78.3)	25 (15.9)	6 (3.8)	3 (1.9)
Morning severity	69 (43.9)	32 (20.4)	35 (22.3)	21 (13.4)
Easily crying	138 (87.9)	16 (10.2)	2 (1.3)	1 (0.6)
Insomnia	113 (72.0)	31 (19.7)	11 (7.0)	2 (1.3)
Lack of appetite	53 (33.8)	10 (6.4)	19 (12.1)	75 (47.8)
Decreased interest in sex	54 (34.4)	13 (8.3)	24 (15.3)	66 (42.0)
Weight loss	140 (89.2)	16 (10.2)	1 (0.6)	0 (0)
Constipation	118 (75.2)	31 (19.7)	7 (4.5)	1 (0.6)
Palpitation	131 (83.4)	22 (14.0)	3 (1.9)	1 (0.6)
Exhaustion	113 (72.0)	38 (24.2)	5 (3.2)	1 (0.6)
Difficulty in thinking	45 (28.7)	14 (8.9)	28 (17.8)	70 (44.6)
Scare capacity	51 (32.5)	13 (8.3)	26 (16.6)	67 (42.7)
Uneasiness	123 (78.3)	29 (18.5)	5 (3.2)	0 (0)
Despair	41 (26.1)	16 (10.2)	32 (20.4)	68 (43.3)
Emotion evoked	119 (75.8)	29 (18.5)	7 (4.5)	2 (1.3)
Hesitation	48 (30.6)	27 (17.2)	37 (23.6)	45 (28.7)
Futility	39 (24.8)	18 (11.5)	41 (26.1)	59 (37.6)
Feeling of living in a void	34 (21.7)	81 (51.6)	42 (26.8)	0 (0)
No value	145 (92.4)	10 (6.4)	1 (0.6)	1 (0.6)
Interest loss	40 (25.5)	10 (6.4)	28 (17.8)	79 (50.4)

## Discussion

The COVID-19 epidemic is one of the most virulent events that has ever threatened health care systems worldwide [8]. The results of this study show that hospital staff were significantly anxious during the pandemic, and their degrees of concern were moderately high. The greatest concerns of health care workers during the COVID-19 pandemic have been found to be infection and the potential consequences of the disease on their health [9]. At the time of writing of this paper, there had been over 3000 confirmed cases among health care workers [2]. The infection rate of medical personnel in Hubei was eight times higher than that of the general population for many reasons. Firstly, hospitals were the focus of confirmed patients; therefore, health care workers were by necessity susceptible to be exposed to the virus as well as to high exposure doses [3]. It has been proved that SARS-CoV-2 is transmitted through respiratory droplets, contact, and fecal-oral contact; even the eye is a possible transmission channel [10]. The generation of aerosols which only existed in hospital wards greatly exacerbated health care workers’ risk of infection [10]. As a result, the perceived risk of being infected was moderately high, and more than 50% (30/54, 55.6%) of respondents had psychological disorders.

Our results showed that relatively high numbers of health care workers experienced moderately high levels of worry during the pandemic, with nurses being more worried than physicians. This phenomenon is reasonable and can be explained by the following causes: (1) the nurses had more and closer contact with patients, and operations such as suctioning and collecting throat swabs exposed them to the disease; and (2) the nurses were generally younger and less educated than the physicians, so they lacked rich clinical experience and had received inadequate mental health education to cope with the difficult situation of the pandemic.

The results of this survey results revealed that the health care workers that came from other cities had higher rates of sleep problems. It is reasonable that the medical staff from outside Hubei Province were not familiar with the particular operation mode in this province compared with local workers. Furthermore, it was inevitable that they would feel fear when they left family and friends, not to speak of going to a dangerous place on their own [11]. Psychological counseling for frontline clinicians should be given attention, especially for those who left their original working environment and assisted during the outbreak in Wuhan.

In general, our research showed that frontline clinicians and clinical medicine students all tended to have higher rates of depression than of anxiety, and more research must be performed to determine the reasons for this finding. There was no significant difference in the rate of psychological disorders between frontline clinicians and medical students (*P*=.85). It was evident that frontline clinicians were at greater risk of infection; however, they had a stronger mentality.

Fortunately, most designated hospitals admitting patients infected with SARS-CoV-2 had established a shift system to allow medical staff to obtain sufficient rest. The government and people in general are making their best efforts to provide adequate backup resources for hospitals. We are hopeful that the psychological problems of medical workers can be alleviated if sufficient attention is given to this issue.

## Abbreviations

SAS: Self-Rating Anxiety Scale
SDS: Self-Rating Depression Scale
