# Correction: Psychological Impact of the COVID-19 Pandemic on Chinese Health Care Workers: Cross-Sectional Survey Study

**DOI:** 10.2196/27596

**Published:** 2021-02-02

**Authors:** Jie Ni, Fang Wang, Yihai Liu, Mingyue Wu, Yan Jiang, Yujie Zhou, Dujuan Sha

**Affiliations:** 1 General Medical Department Nanjing Drum Tower Hospital the Affiliated Hospital of Nanjing University Medical School Nanjing China; 2 Department of Emergency Nanjing Drum Tower Hospital the Affiliated Hospital of Nanjing University Medical School Nanjing China; 3 Department of Cardiology Nanjing Drum Tower Hospital Clinical College of Nanjing Medical University Nanjing China; 4 Department of Education Nanjing Drum Tower Hospital the Affiliated Hospital of Nanjing University Medical School Nanjing China

In “Psychological Impact of the COVID-19 Pandemic on Chinese Health Care Workers: Cross-Sectional Survey Study” (JMIR Ment Health 2021;8(1):e23125) the authors noted four errors.

In the originally published manuscript, author Yujie Zhou was listed twice. The duplicate instance of this author has been removed in the corrected version.

The originally published manuscript had no equal contribution footnote. This has been corrected so that authors Jie Ni, Fang Wang, and Yihai Liu are noted as equal contributors.

Affiliations for all authors have also been corrected. The full list of authors and affiliations in the originally published version of the paper was:

Jie Ni, MD; Fang Wang, MD; Yihai Liu, PhD; Mingyue Wu, MD; Yan Jiang, MD; Yujie Zhou, MD; Yujie Zhou, MD; Dujuan Sha, MD

Nanjing Drum Tower Hospital, Nanjing, China

Taking into account all of the above corrections, the full list of authors and affiliations has been corrected to:

Jie Ni^1*^, MD; Fang Wang^2*^, MD; Yihai Liu^3*^, MD; Mingyue Wu^3^, MD; Yan Jiang^4^, MD; Yujie Zhou^4^, MD; Dujuan Sha^1^, MD

^1^General Medical Department, Nanjing Drum Tower Hospital, the Affiliated Hospital of Nanjing University Medical School, Nanjing, China

^2^Department of Emergency, Nanjing Drum Tower Hospital, the Affiliated Hospital of Nanjing University Medical School, Nanjing, China

^3^Department of Cardiology, Nanjing Drum Tower Hospital, Clinical College of Nanjing Medical University, Nanjing, China

^4^Department of Education, Nanjing Drum Tower Hospital, the Affiliated Hospital of Nanjing University Medical School, Nanjing, China

^*^these authors contributed equally

The corresponding author address has also been corrected. In the originally published paper, the corresponding author was:

Yujie Zhou, MD

Nanjing Drum Tower Hospital

Zhongshan Road 321

Nanjing

China

Phone: 1 8206299821

Email: jiji218506@sina.com

This has been corrected to:

Dujuan Sha, MD

General Medical Department

Nanjing Drum Tower Hospital

the Affiliated Hospital of Nanjing University Medical School

Zhongshan Road 321

210000

Nanjing

China

Phone: 86 13951980866

Email: tbwen0912@126.com

The correction will appear in the online version of the paper on the JMIR Publications website on February 2, 2021, together with the publication of this correction notice. Because this was made after submission to PubMed, PubMed Central, and other full-text repositories, the corrected article has also been resubmitted to those repositories.

